# Private Hospitals and the Trained Nurse's Responsibility

**Published:** 1921-03-12

**Authors:** 


*To the Editor of* The Hospital.

### Private Hospitals and the Trained Nurse's Responsibility.

*To the Editor of* The Hospital.

Sir,—I should like to thank "Ierne" for the outspoken articles on private hospitals which have appeared in your paper (February 5 and 26). Those of us who have had friends in nursing homes, either as patients or as nurses, know that the statements are not exaggerated. Legislation on the matter is long overdue. Patients have for years been paying heavily (in every sense of the words) for the privilege of being nursed in uncomfortable and unhygienic surroundings by untrained or half-trained nurses. And though charges have increased largely, conditions remain much the same. But legislation tarries, and patients are suffering now. Trained nurses could take action at once, though they cannot remedy the evil entirely. Private hospitals have nearly always one or more trained nurses on the staff. If these nurses would refuse to stay where the conditions were hopelessly bad, the proprietors would be entirely dependent on unskilled help. They would then be driven either to alter their methods or to give up their speculation altogether. There cannot be many doctors who would risk their patients' lives by allowing them to go to a private hospital with no trained nurse on its staff.—I remain, yours faithfully,

